# Fast and accurate correction of optical mapping data via spaced seeds

**DOI:** 10.1093/bioinformatics/btz663

**Published:** 2019-09-03

**Authors:** Leena Salmela, Kingshuk Mukherjee, Simon J Puglisi, Martin D Muggli, Christina Boucher

**Affiliations:** 1 Department of Computer Science, Helsinki Institute for Information Technology HIIT, FI-00014 University of Helsinki, Helsinki 00100, Finland; 2 Department of Computer and Information Science and Engineering, University of Florida, Gainesville, FL 32611, USA; 3 Department of Computer Science, Colorado State University, Fort Collins, CO 80523, USA

## Abstract

**Motivation:**

Optical mapping data is used in many core genomics applications, including structural variation detection, scaffolding assembled contigs and mis-assembly detection. However, the pervasiveness of spurious and deleted cut sites in the raw data, which are called Rmaps, make assembly and alignment of them challenging. Although there exists another method to error correct Rmap data, named cOMet, it is unable to scale to even moderately large sized genomes. The challenge faced in error correction is in determining pairs of Rmaps that originate from the same region of the same genome.

**Results:**

We create an efficient method for determining pairs of Rmaps that contain significant overlaps between them. Our method relies on the novel and nontrivial adaption and application of spaced seeds in the context of optical mapping, which allows for spurious and deleted cut sites to be accounted for. We apply our method to detecting and correcting these errors. The resulting error correction method, referred to as Elmeri, improves upon the results of state-of-the-art correction methods but in a fraction of the time. More specifically, cOMet required 9.9 CPU days to error correct Rmap data generated from the human genome, whereas Elmeri required less than 15 CPU hours and improved the quality of the Rmaps by more than four times compared to cOMet.

**Availability and implementation:**

Elmeri is publicly available under GNU Affero General Public License at https://github.com/LeenaSalmela/Elmeri.

**Supplementary information:**

Supplementary data are available at *Bioinformatics* online.

## 1 Introduction

Optical mapping is a process in which DNA is first isolated, denaturated and fragmented, restriction enzymes are then applied to the fragmented DNA to further cut it at prescribed restriction sites, before finally the resulting DNA is imaged to capture the relative order and size of the fragments between the cut sites (Dimalanta *et al.*, 2004; Samad *et al.*, 1995). The ordered lists of fragment sizes are the result of this process and are referred to as *Rmaps*. Rmaps are analogous to sequence reads in the context of genome sequencing, and, as with genome sequencing, the optical mapping process is repeated for all DNA molecules in a single sample. This leads to there being overlap between pairs of Rmaps, which are then used to error correct or assemble the set of Rmaps produced by a single experiment. Rmap data spans genomic regions that are significantly longer than short read sequence data—200 kbp versus 250 bp—but cheaper than long read data, making it a viable option to reference-based assembly (Lin *et al.*, 1999), identification of mis-assembled regions in draft genomes (Muggli *et al.*, 2015), structural variation detection (Teague *et al.*, 2010) and *de novo* assembly of large genomes (Beier *et al.*, 2017; Daccord *et al.*, 2017; Dong *et al.*, 2013; Ganapathy *et al.*, 2014; Jarvis *et al.*, 2017; Vij *et al.*, 2016).

Due to the fragile nature of the DNA and the inexactness of restriction enzymes, Rmaps are prone to having added or deleted cut sites. When Rmaps are viewed as a sequence of fragment sizes, added and deleted cut sites merge or split fragments and thus appear as insertions and deletions. In addition, the measurement of the fragment is error prone and likely to differ from the true length of the DNA fragment. These errors make finding pairs of overlapping Rmaps challenging.

Previous approaches, such as our earlier Rmap error correction method cOMet (Mukherjee *et al.*, 2018), use *k*-mers as seeds to detect whether two Rmaps have significant overlap. More specifically, as Rmaps are sequences of fragment lengths, *k*-mers are defined as *k* consecutive fragment lengths observed in an Rmap. Thus, these tools compare all *k*-length subsequences of one Rmap with every other Rmap to find those that have a significant number in common; those that do are deemed to have significant alignments. Due to the presence of errors in the Rmaps, *k* has to be very small (less than 5 where the average Rmap length is 20 fragments long), resulting in a method that has high sensitivity but low specificity. Extra filtering has to be done in order to increase the specificity at the expense of running time.

In this paper, we view Rmaps as line segments, where cut sites are points on that line. In this representation added and deleted cut sites behave like mismatches as the cut site is either present or not on the line segment. Furthermore, this representation offers another natural way of extending the notion of *k*-mers to optical mapping. Instead of using *k* consecutive fragments as a seed, we can use a fixed length subsegment of the line as a seed. Here, we call these seeds *ℓ*-mers. We further extend this definition to (*ℓ*, *k*)-mers which require the seeds to have at least *k* fragments and spaced (*ℓ*, *k*)-mers, which allow for gaps in the seeds that account for the mismatches caused by added and deleted cut sites.

The concept of spaced (*ℓ*, *k*)-mers is used for error correction of Rmap data. Our previous method, referred to as cOMet (Mukherjee *et al.*, 2018), is currently the only available method for error correction of Rmap data. It can correct a large number of added and deleted cut sites but requires almost 10 CPU days to error correct Rmap data from the human genome. Thus, for even moderately large sized genomes, high performance computing resources are needed. In this paper, we give an error correction algorithm that is based on spaced (*ℓ*, *k*)-mers. We refer to our method as Elmeri, and demonstrate that it is 16 times faster than cOMet (Mukherjee *et al.*, 2018) on the human data. In addition, Elmeri gives better quality results. We aligned the original (uncorrected) data, the data corrected by cOMet and the data corrected by Elmeri to the error-free reference genome wide optical map and found that the mean improvement on alignment scores of Rmaps corrected by Elmeri is more than four times that of cOMet.

Lastly, we mention that the concept of spaced (*ℓ*, *k*)-mers may be of interest in other applications of optical mapping data such as alignment of Rmaps and assembly of Rmaps into genome wide optical maps where similar Rmaps need to be found. Our experiments show that spaced (*ℓ*, *k*)-mers can have more than double recall with a similar precision as compared to *k*-mers in identifying similar Rmaps.

## 2 Related work

### 2.1 Algorithms for optical maps

Several methods have been developed to analyze optical mapping data—either alone or in conjunction with sequence data. The most closely related work to the one presented in this paper is our previous method for error correction of Rmap data (Mukherjee *et al.*, 2018), which we discussed in the introduction. AGORA (Lin *et al.*, 2012) and misSEQuel (Muggli *et al.*, 2015) aim at using optical mapping data to help in determining or preventing mis-assembled contigs. AGORA performs sequence assembly guided by optical maps by comparing *in silico* digested contigs (which correspond to a path in the de Bruijn graph) to the optical map. If the paths do not agree then the path is discarded as incorrect. misSEQuel (Muggli *et al.*, 2015) assembles sequence data, aligns *in silico* digested contigs to optical maps using Twin (Muggli *et al.*, 2014) and then deciphers which contigs are misassembled based on the alignment. Most-recently Pan *et al.* (2019) presented OMGS, which is an optical mapping based genome scaffolder.

Both Valouev *et al.* (2006a) and Nagarajan *et al.* (2008) use dynamic programming to compute the best alignment between pairs of optical mapping sequences, where these sequences can be Rmaps and/or genome wide optical maps. Valouev *et al.* computes the best alignment using a dynamic programming based scoring scheme similar to the Needleman–Wunsch algorithm (Needleman and Wunsch, 1970). Their scoring function is defined as a log likelihood ratio test that takes into account the various errors prevalent in the optical map data. SOMA (Nagarajan *et al.*, 2008) aligns assembled contigs to a genome-wide optical map using a dynamic programming algorithm that is optimized by using a different scoring function that imposes a fixed cost penalty on added and deleted cut sites, and a chi-squared function to penalize for sizing errors.

In the past several years, new data structures and algorithms have been applied to optical map alignment to create Twin (Muggli *et al.*, 2015), OMBlast (Leung *et al.*, 2017), Maligner (Mendelowitz *et al.*, 2016) and Kohdista (Muggli *et al.*, 2018). OMBlast modifies the seed-and-extend approach used in BLAST (Altschul *et al.*, 1990) for finding alignments in optical mapping data. Maligner provides two modes of alignment: an efficient, sensitive dynamic programming implementation that scales to large eukaryotic genomes, and a faster index based implementation for finding alignments with unmatched sites in the reference but not the query. Twin (Muggli *et al.*, 2014) uses an FM-index for aligning contigs to a genome-wide optical map. The FM-index search is modified to allow sizing errors but Twin is unable to account for added and deleted cut sites. Kohdista (Muggli *et al.*, 2018) formulates the alignment problem as automaton path searching and thus tolerates both sizing errors and added and deleted cut sites.

### 2.2 Seeds and spaced seeds

Homology search algorithms, such as BLAST (Altschul *et al.*, 1990) pervade bioinformatics. Initially, determining whether two sequences were homologous or not was based on pairwise alignment of the two sequences using dynamic programming algorithms with quadratic time complexity. Yet, computing the alignment between pairs of sequences is infeasible with large sequence sets as the number of sequence pairs also increases quadratically. Computing and comparing a set of seeds remedied this problem. The main idea is that homologous sequences have well-conserved regions which are very similar in all sequences and thus, identical seeds can be found in these regions. For example BLAST (Altschul *et al.*, 1990) uses exact matches of *k*-length sequences as ‘seeds’ and extends the seed matches to longer alignments containing them.

The introduction and application of spaced seeds represented another major advance in homology search (Burkhardt and Kärkkäinen, 2003; Choi *et al.*, 2004; Ilie and Ilie, 2007; Ma *et al.*, 2002). Spaced seeds extend the idea of an exact seed: a spaced seed is *k* discontinuous nucleotide matches, where there exists a preset number of wildcard positions that match any nucleotide (Keich *et al.*, 2004). PatternHunter (Ma *et al.*, 2002) introduced the concept of *optimized spaced seed*, where the relative positions of the *k* nucleotides are optimized in advance. This extension allowed PatternHunter to significantly increase its sensitivity over BLAST. Independently, Buhler *et al.* (2005), Ma *et al.* (2002) and Brejová *et al.* (2003) noticed that increasing the number of spaced seeds will increase sensitivity. In practice, this concept of comparing seeds has been shown to have high sensitivity and specificity for homology search even when the spaced seeds are not optimized (Li *et al.*, 2004; Ma *et al.*, 2002).

Spaced seeds are not confounded by mismatches and thus, are effective when there exist regions that are well-conserved and uninterrupted by insertions or deletions (*indels*). However, when the frequency of indels increases, the length of the seed or the number of common seeds becomes low enough that it is no longer effective for sensitive and specific detection of alignment, which has been witnessed in long read alignment (Chaisson and Tesler, 2012).

## 3 Background and definitions

### 3.1 Rmaps and optical mapping

From a computational perspective, optical mapping takes in a DNA sequence and a restriction enzyme that cuts the DNA at a unique restriction site, and returns a sequence (or array) of fragment sizes R, i.e. where R[i] is the number of nucleotides between the (i−1)th and *i*th cut site. Analogous to short read sequencing, the method is commonly applied to multiple DNA molecules from the same individual. The result is millions of fragmented DNA molecules that overlap. The raw data resulting from the process is referred to as *Rmaps*—whose analogue are sequence reads in the context of genome sequencing.

We denote an Rmap R as $\left[r_{1},r_{2},\ldots ,r_{n}\right]$. We refer to the *size* of R as the number of fragments, i.e. *n* is the size of *R*, and the *length* of R as $|R|=\sum_{i=1}^{n}r_{i}$. For example, given a DNA sequence CGCGTCGCGAATATCGCGTTAATAATAACGCGACGCG and a restriction site sequence CGCG, the corresponding Rmap is R=[5,9,14,5] assuming that the restriction enzyme cuts between the center G and C in the restriction site.

**Fig. 1.**
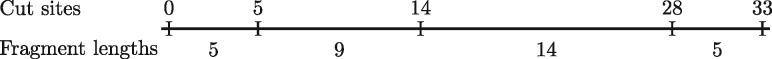
The cut site representation (above) and the fragment length representation (below)

Equivalently R can be viewed as a sequence of cut site locations in the DNA. More formally, optical mapping takes in a DNA sequence A[1..a] and a restriction sequence B[1..b], and produces an array (string) of integers $C=[c_{1},c_{2},\ldots ,c_{n+1}]$, such that $c_{i}=j-1$ if and only if the substring A[j..j+b−1]=B is the *i*th occurrence of B in A. Without loss of generality we assume that $c_{1}=0$ i.e. the first cut site occurs at A[1…b]. This definition of Rmaps allows us to visualize an Rmap as a line segment from 0 to |R| where each cut site *c_i_* is a point on the line. We note that $r_{i}=c_{i+1}-c_{i}$ and $c_{i}=\sum_{j=1}^{i-1}r_{i}$. It will be more natural to describe some of further definitions using this *cut site representation* rather than the former *fragment size representation*. For our running example R=[5,9,14,5], the corresponding cut site representation is C=[0,5,14,28,33]. Figure 1 illustrates the two representations. We will prompt the reader when we use the cut site representation; otherwise, it can be assumed that we will use the fragment size representation.

Here, for simplicity, we have used small examples and integer values for the fragment sizes. In practice fragment sizes are given in 1000 bp (1 kbp) and real numbers. For example, on the real human Bionano dataset (Shi *et al.*, 2016) cut sites occur once every 10 kbp and the average Rmap covers 200 kbp. Also, we note that in practice high-throughput optical mapping technologies (e.g. Bionano) use enzymes that nick the DNA rather than cutting it to decrease the error rate of the resulting data.

Nonetheless, Rmaps are highly error prone— the majority of errors are characterized as one of the following three types: (i) sizing error, (ii) deleted cut sites and (iii) added cut sites. Sizing error occurs from inability of estimating the size of the fragments exactly. For example, if 10 bp exists between two cut sites, the optical mapping process may output the size to be 12 bp, resulting in a 2 bp error in the size of the fragment. Secondly, restriction enzymes can end up deleting a cut site, which results in the two neighboring fragments being merged into one. In the above example, if the enzyme misses the second cut site, the resulting Rmap would be [14,14,5]. Lastly, due to the fragile nature of DNA, the DNA molecule can break spontaneously at a location where a restriction site is not present. This causes a fragment to be split into two. For example, the third fragment in our example could be split into two fragments, 8 and 6, resulting in the Rmap [5,9,6,8,5].

### 3.2 *k*-mers, *ℓ*-mers and spaced (*ℓ*, *k*)-mers

**Fig. 2.**
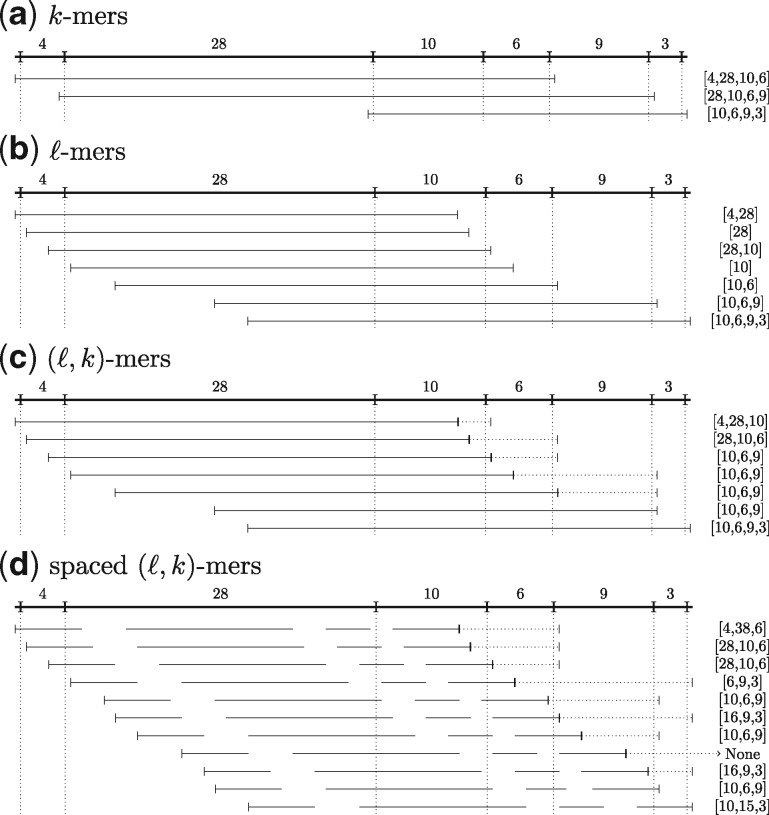
Extraction of (**a**) *k*-mers for *k *=* *4, (**b**) *ℓ*-mers for ℓ=40, (**c**) (*ℓ*, *k*)-mers for ℓ=40 and *k *=* *3 and (**d**) spaced (*ℓ*, *k*)-mers for ℓ=40, *k *=* *3 and spacing pattern *S *=* *1111110000111111111111111000111100111111 from the Rmap [4,28,10,6,9,3]. For (*ℓ*, *k*)-mers and spaced (*ℓ*, *k*)-mers we use dotted lines to show the extension beyond *ℓ* positions to include at least *k* fragments. For spaced (*ℓ*, *k*)-mers missing line segments denote spaces in the spacing pattern. The extracted *k*-mers, *ℓ*-mers, (*ℓ*, *k*)-mers and spaced (*ℓ*, *k*)-mers are shown on the right. To extract all *k*-mers from an Rmap, we first extract the *k*-mer containing the *k* leftmost fragments. To get the next *k*-mer, the leftmost fragment is dropped and the next fragment on the right is added. To extract all *ℓ*-mers and (spaced) (*ℓ*, *k*)-mers we first consider the *ℓ*-length subsegment of the line positioned at the leftmost position. To get the next *ℓ*-mer or (spaced) (*ℓ*, *k*)-mer, the subsegment is shifted to the right until a cut site enters or exits (the solid part of) the subsegment

A *k*-mer of an Rmap R is a *k*-length subsequence of fragment sizes of R. For example, given an Rmap R=[4,28,10,6,9,3] and *k *=* *4, three *k*-mers, [4,28,10,6], [28,10,6,9] and [10,6,9,3], can be extracted from R as shown in Figure 2a.

For simplicity of explanation, we consider the cut site representation of an Rmap in this subsection. We define an *ℓ*-mer as a sequence of all cut sites of C contained within an interval [p,p+ℓ−1] for some $0\leq p\leq c_{n+1}-\ell +1$. From a more intuitive perspective, if we illustrate an Rmap as a line segment starting at 0 and ending at position $c_{n+1}=|R|$ where the cut sites are points on that line segment, then an *ℓ*-mer corresponds to any consecutive sequence of elements of C such that they can be fully contained within an *ℓ*-length subsegment and all cut sites within the *ℓ*-length subsegment are included. This is illustrated in Figure 2b. In our method we use the fragment size representation of *ℓ*-mers: If the cut site representation of an *ℓ*-mer is $\left[c_{i},\ldots ,c_{i+x}\right]$ then the corresponding fragment size representation is $\left[r_{i},\ldots ,r_{i+x-1}\right]$, where $r_{j}=c_{j+1}-c_{j}$.

To extract all different *ℓ*-mers from an Rmap, we start by positioning the line subsegment at the leftmost position. The next position for the line subsegment is obtained by moving it to the right until either the leftmost cut site currently within the line subsegment drops outside the line subsegment or the next cut site on the right enters the line subsegment. Figure 2b illustrates this process for Rmap R=[4,28,10,6,9,3] when ℓ=40.

Some *ℓ*-mers can have very few fragments, which is problematic in practice since these are usually not unique in the genome. To correct for this, we define an (*ℓ*, *k*)-mer of Rmap *R* by computing all *ℓ*-mers of *R* and inserting fragments from the right until there exists at least *k* fragments. Going back to our line segment illustration, this corresponds to extending the *ℓ*-length line subsegment to the right until at least *k* fragments are fully contained in it. We note that there can be more than *k* fragments if the *ℓ*-mer contains more than *k* fragments. Figure 2c illustrates the extraction of (*ℓ*, *k*)-mers for ℓ=40 and *k *=* *3. (*ℓ*, *k*)-mers are a generalization of *k*-mers and *ℓ*-mers: (0,k)-mers are equivalent to *k*-mers and (ℓ,0)-mers are equivalent to *ℓ*-mers.

We now extend the definition of (*ℓ*, *k*)-mers to spaced (*ℓ*, *k*)-mers. Spaced (*ℓ*, *k*)-mers are defined by *ℓ*, *k* and a spacing pattern *S* of length *ℓ*. A spacing pattern is a sequence of 0 and 1 s, where 0 s denote gaps, and 1 s denote solid parts. Again we consider the cut site representation of an Rmap. We align the spacing pattern to an *ℓ*-mer corresponding to the interval [p,p+ℓ−1] for some 0≤p≤|R|−ℓ+1. Then we construct a modified Rmap $C\prime =[c\prime_{1},\ldots ,c\prime_{n\prime +1}]$ in cut site representation by removing any cut site from *C* that falls within a gap in the spacing pattern. The corresponding modified Rmap $R\prime =[r\prime_{1},\ldots ,r\prime_{n\prime }]$ in fragment size representation can now be constructed by setting $r\prime_{i}=c\prime_{i+1}-c\prime_{i}$. The spaced (*ℓ*, *k*)-mer is then defined as the (*ℓ*, *k*)-mer extracted from R′ at position *p*. Going back to our line segment illustration, all spaced (*ℓ*, *k*)-mers can be extracted from an Rmap by first aligning the spacing pattern to the leftmost position of the line segment and extracting the first spaced (*ℓ*, *k*)-mer. The spacing pattern is then shifted to the right until a cut site exits or enters the solid part of the spacing pattern and the next spaced (*ℓ*, *k*)-mer is extracted. We repeat the shifting of the spacing pattern until its end is shifted beyond the line segment. Figure 2d illustrates this process for ℓ=40, *k *=* *3 and *S *=* *1111110000111111111111111000111100111111.

As mentioned earlier, in practice fragment sizes are in thousands of base pairs and so typical values of *ℓ* are in tens of thousands of base pairs. Thus it is impractical to have a spacing pattern of length *ℓ*. Instead in practice each bit of the spacing pattern spans ℓ/|S| base pairs.

## 4 Methods

**Fig. 3.**
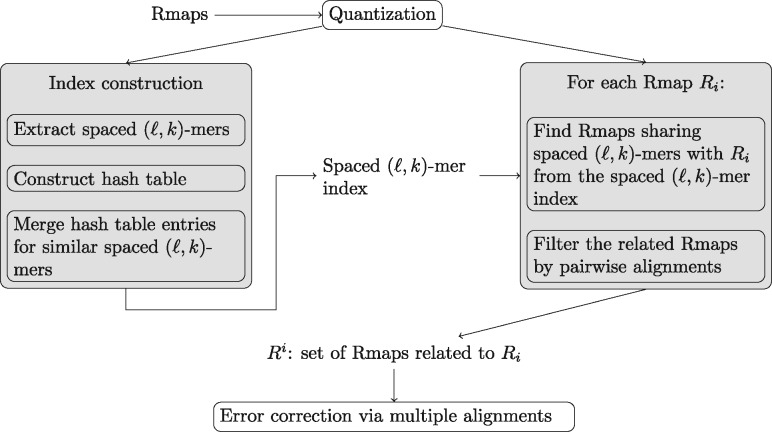
Overview of the error correction process in Elmeri

The input to our method is a set (Actually, the input is a multi-set since we allow repetition.) of Rmaps $R=\{R_{1},\ldots ,R_{m}\}$. First, we create a spaced (*ℓ*, *k*)-mer index that maps spaced (*ℓ*, *k*)-mers to the Rmaps in which they occur in. The index is further refined by merging entries for similar spaced (*ℓ*, *k*)-mers. Then, we use this index to find a sets of Rmaps that share spaced (*ℓ*, *k*)-mers for a given Rmap *R_i_* and thus, are likely to originate from the same genomic area. We repeat this for all Rmaps which results in a set of Rmaps for each *R_i_*. Next, we filter each set by aligning each Rmap pairwise to *R_i_* and keeping only those that align well enough. Lastly, we create a multiple alignment of each set of Rmaps and correct them towards the consensus of the multiple alignment. The overview of our method is shown in Figure 3. Each of the steps of our method is discussed in detail in the following subsections. Further, we present a method for optimizing the spacing pattern used in the spaced (*ℓ*, *k*)-mer index.

### 4.1 Finding sets of related Rmaps

First, we quantize the fragment lengths in each Rmap to account for the sizing error. We use bins of fixed length *b* for the quantization (by default *b *=* *1000), i.e. if the original fragment length is *x* then the quantized length is ⌊x/b⌋.

Next, we extract spaced (*ℓ*, *k*)-mers from each Rmap, and use a hash table to map each spaced (*ℓ*, *k*)-mer to the Rmaps that contain it. Given a spaced (*ℓ*, *k*)-mer *M*, we denote the corresponding set of Rmaps as R(M), i.e. $R(M)=\{R_{i}\in R|M\;\text{occurs}\;\text{in}\;R_{i}\}$.

Most fragments originating from the same region of the genome are quantized to the same value. However, when the correct fragment length is close to the boundary of two quantization bins, the estimated fragment lengths can fall into either bin. Therefore, to find related Rmaps more effectively, we merge the sets of Rmaps corresponding to similar spaced (*ℓ*, *k*)-mers.

Given two spaced (*ℓ*, *k*)-mers *M*_1_ and *M*_2_ with the same number of fragments, we define their distance as follows:

$$\text{dist}(M_{1},M_{2})=\sum_{i=1}^{\left|M_{1}\right|}|M_{1}[i]-M_{2}[i]|.$$

We then set a similarity threshold $t_{\text{sim}}$ and extend the set of Rmaps corresponding to a spaced (*ℓ*, *k*)-mer:

$$R\prime (M)=\underset{M\prime |\text{dist}(M,M\prime )\leq t_{\text{sim}}}{⋃}R(M\prime ).$$

In practice the above procedure is inefficient with respect to memory usage because many Rmaps are replicated several times with small variations. Thus, we implemented a heuristic merging procedure for the Rmap sets as follows. For each set R′(M) we keep a counter *i_M_* initialized to 1 indicating how many spaced (*ℓ*, *k*)-mers have been merged to it. We iterate through all spaced (*ℓ*, *k*)-mers in the index twice. For each spaced (*ℓ*, *k*)-mer *M*, we find the spaced (*ℓ*, *k*)-mers M′ for which dist(M,M′)=1. If $i_{M}+i_{M\prime }-1\leq t_{\text{sim}}$ then we set both R′(M) and R′(M′) to R′(M)∪R′(M′) and both *i_M_* and $i_{M\prime }$ equal to $i_{M}+i_{M\prime }$. This process guarantees that the Rmap sets for two spaced (*ℓ*, *k*)-mers with distance higher than the similarity threshold are never merged. However, Rmap sets for some spaced (*ℓ*, *k*)-mers that are similar enough might not get merged if they have been previously merged with other sets.

To find sets of related Rmaps, we iterate through the set $\left\{R_{1},\ldots ,R_{m}\right\}$. Let *R_i_* be the current Rmap. We extract all spaced (*ℓ*, *k*)-mers from *R_i_*, query the spaced (*ℓ*, *k*)-mer index for each spaced (*ℓ*, *k*)-mer and count the number of spaced (*ℓ*, *k*)-mers each Rmap shares with *R_i_*. Using these counts, we select the *N* Rmaps that share the greatest number of spaced (*ℓ*, *k*)-mers with *R_i_* to be the set of *related Rmaps* for *R_i_*. We denote this set of related Rmaps of *R_i_* as *R^i^*. Lastly, we note that *N* should be roughly equal to the coverage of the Rmap set, and set the default value of *N* to be 64.

**Fig. 4.**
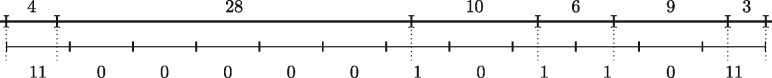
Transforming the Rmap [4,28,10,6,9,3] to a binary string. The block size in this example is 5

As our experiments in Section 5.2 show, the spaced (*ℓ*, *k*)-mer index sometimes return related Rmaps that do not originate from the same genomic area as the current Rmap *R_i_*. Therefore, we filter out unrelated Rmaps from *R^i^* using the following procedure. First, we transform Rmaps into binary strings by using the cut site representation and considering each Rmap as a line segment where each cut site defines a point on the line. We divide each line segment into blocks of size *B* (by default 2000), and transform them into binary strings as follows: if a block contains one or more cut sites then we add as many 1 s as there are cut sites; otherwise, we add a 0. See Figure 4 for an example. We note that this representation is ambiguous with regard to the block boundaries. However, we are concerned with determining which cut sites originate from the same cut site in the genome and thus block boundaries need not be unambiguous.

Next, we align each (transformed) Rmap in *R^i^* pairwise against *R_i_* using a variant of the Needleman-Wunsch algorithm (Needleman and Wunsch, 1970). When aligning the binary strings, we consider the 0 and 1 s to be normal characters. If the alignment contains a gap, this is denoted by a separate gap character, ‘-’. We allow free gaps in the beginning and end of the alignment for both Rmaps allowing us to find prefix-suffix overlaps between them. The binary strings are sparse with only a few 1 s. With uniform edit costs, the algorithm tends not to align the 1 s (i.e. cut sites) because the much more abundant 0 s are easily aligned with each other. Since aligning the cut sites is crucial in our application, we set the alignment costs so that any error involving a cut site costs $(c_{0}+c_{1})/c_{1}$, where *c*_0_ (*c*_1_) is the number of 0 s (1 s) in all binary strings participating in the alignment, and all other errors have a cost of 1. We assume that Rmaps should not have more than 40% added and missing cut sites and thus, an alignment of two Rmaps should not have more than 80% added and added cut sites taking into account errors in both Rmaps. This is a conservative assumption as Li *et al.* (2016) estimate the average digestion rate of an Rmap fragment to be 0.8. Therefore, if the number of edits exceeds 0.8 times the number of cut sites in the Rmap having less cut sites, the related Rmap is removed from *R^i^*.

### 4.2 Multiple alignment based correction of Rmaps

Next, we construct a multiple alignment for *R_i_* and the filtered *R^i^*. We use the same transformation of Rmaps to binary strings as above and then proceed to compute a heuristic multiple alignment for the binary strings. *R_i_* is set as the initial consensus of the alignment. The related Rmaps are then aligned against the consensus consecutively using the same algorithm as for the pairwise alignments above. After each pairwise alignment, the consensus is updated accordingly. If there is a deletion in the newly aligned binary string, a gap symbol is added to that sequence. If there is an insertion in the newly aligned binary string, we add a new column to the multiple alignment at that position and update those binary strings that are already aligned to include the gap. Then we compute for each column in the multiple alignment the most prevalent symbol or gap which then becomes the consensus at that position.

Lastly, we determine if there exists any Rmaps in the multiple alignment that contain a substantial amount of added and/or deleted cut sites as compared to the consensus—indicating that they do not originate from the same genomic region. Hence, we remove any Rmaps that have more than 3 extra cut sites or more than 40% deleted cut sites as compared to the consensus. After this filtering step, we recompute the multiple alignment for all the remaining Rmaps.

Once the multiple alignment for an Rmap *R_i_* and its related Rmaps has been computed, we are ready to correct the Rmaps in the alignment. We require that at least five Rmaps participate in the alignment to proceed to the correction phase.

To get an accurate estimate of the fragment lengths in the consensus we retrieve the original unquantized Rmaps and compute the average of the fragment lengths for all Rmaps that have the fragment in question (i.e. both flanking cut sites have to be present in the Rmap). The aligned Rmaps can then be corrected by extracting from the consensus Rmap the part where that Rmap aligns to.

For high coverage datasets, the above method builds multiple alignments for the same genomic region excessively many times. Since all Rmaps in the alignment are corrected, many Rmaps are corrected more times than is necessary. Therefore, we count how many times each Rmap has already been corrected, and when iterating over the multiset of Rmaps, we skip an Rmap if it has already been corrected many times. In practice, we found a threshold of five corrections to be sufficient for a good overall correction result.

### 4.3 Optimizing the spacing pattern

The accuracy of the spaced (*ℓ*, *k*)-mer index depends on the choice of the spacing pattern. In our experiments we mostly use *ℓ* = 80 kbp and quantization constant *b *=* *1 kbp. The spacing pattern had 80 bits and thus, each bit represents a 1 kbp region. For evaluating the performance of the spaced (*ℓ*, *k*)-mer index using a given spacing pattern we use a small dataset with 2000 Rmaps simulated from the *E.coli* genome. We evaluate the precision and recall of the index on this dataset as detailed in Section 5.2 and use F-score, which is the harmonic mean of precision and recall, to evaluate the fitness of the spacing patterns. Exhaustive enumeration of all 80 bit spacing patterns is infeasible and thus we use a simulated annealing algorithm to optimize the spacing pattern. We initialize the algorithm with a random spacing pattern where the probability of both 0 and 1 is 0.5. In each round we then choose a random bit and flip it. If the new spacing pattern is better than the previous one, it always becomes the current spacing pattern. If the new spacing pattern is worse than the previous one, we still accept it with a probability depending on the current temperature as in a simulated annealing algorithm.

## 5 Experimental results

In this section, we first compare indexing schemes which use *k*-mers, *ℓ*-mers, (*ℓ*, *k*)-mers and spaced (*ℓ*, *k*)-mers to show the advantage of the spaced (*ℓ*, *k*)-mers. Then, we compare the performance of Elmeri to that of cOMet (Mukherjee *et al.*, 2018).

### 5.1 Data

We performed experiments on both simulated and real Bionano datasets. We used the simulated Bionano Rmap datasets from Mukherjee *et al.* (2018), which are generated from the *E.coli* K-12 substr. MG1655 genome with OMSim (Miclotte *et al.*, 2017). Default parameters with enzyme BspQI were used with varying rates at which additional and missing cut sites were introduced. The number of additional cut sites was varied from 0.5 to 5 per 100 kbp and the percentage of missing cut sites was varied from 5 to 25%. In total eight different datasets were produced that have a varying number of Rmaps, e.g. between 123 251 and 157 743 Rmaps. We collectively refer to these eight datasets as Ecoli1.

In addition, we used the simulated Rmap *E.coli* data from Mukherjee *et al.* (2018) which was generated by first constructing error free Rmaps from the *E.coli* K-12 substr. MG1655 genome and then introducing added and missing cut sites and sizing error according to the error model by Li *et al.* (2016). The resulting dataset consists of 2504 error-free Rmaps and 2504 Rmaps, which contain 7485 missing cut sites and 554 additional cut sites. We refer to this dataset as Ecoli2.

Lastly, we performed experiments using the human Bionano Rmap dataset produced by Shi *et al.* (2016), which consists of 793 199 Rmaps and *Anabas testudineus* (climbing perch) genome generated for the Vertebrate Genome Project, which consists of 3 121 480 Rmaps. Table 1 summarizes the datasets used in the experiments.

**Table 1.** Datasets used in the experiments

Dataset	Genome	Genome size	Number of Rmaps
Ecoli1	*E.coli* K-12 MG 1655	4.6 Mbp	123 251–157 743
Ecoli2	*E.coli* K-12 MG 1655	4.6 Mbp	2504
Human	Chinese individual (HX1)	3.2 Gbp	793 199
AnaTes	*Anabas testudineus*	0.66 Gbp	3 121 480

*Note*: The Ecoli1 dataset contains eight simulated *E.coli* datasets with varying error rates and thus also the number of Rmaps varies. The genome size of *A.testudineus* is an estimate since there exists no reference genome.

### 5.2 Comparison of the indexing schemes

For evaluating the indexing schemes, we used a subset of 2000 Rmaps with at least 10 fragments from the Ecoli1 dataset which contains 1.0 additional cut site per 100 kbp and 15% missing cut sites. We used a small subset instead of the full dataset, to quickly explore a large parameter space of the proposed method. Because the Rmaps are simulated, we know their genomic positions. We classified two Rmaps as related if their genomic positions overlap by at least 100 kbp and by at least 7 fragments. For a predicted set of related Rmaps we can now compute following statistics: (i) true positives (TP), i.e. number of Rmap pairs which are predicted to be related and are also actually related, (ii) false positives (FP), i.e. number of Rmap pairs which are predicted to be related but are actually not and (iii) false negatives (FN), i.e. number of Rmap pairs which are predicted not to be related but are actually related. Based on these we computed precision and recall: Precision=TP/(TP+FP), Recall=TP/(TP+FN).

We compared the following indexing schemes:

- *k*-mers
- *k*-mers with merging of similar *k*-mers
- *ℓ*-mers with merging of similar *ℓ*-mers
- (ℓ,5)-mers with merging of similar (ℓ,5)-mers
- Spaced *ℓ*-mers with merging of similar spaced *ℓ*-mers
- Spaced (ℓ,5)-mers
- Spaced (ℓ,5)-mers with merging of similar spaced (ℓ,5)-mers

All indexes using spaced (*ℓ*, *k*)-mers used the spacing pattern 11111111110001110110010010011101001110001010010100001010011000010111100000001100. In all cases two Rmaps are considered related if they share at least two *k*-mers/*ℓ*-mers/(ℓ,5)-mers. To obtain various precision-recall measurements, we varied the value of *k* or *ℓ* for each scheme. Thus, *k* was varied from 3 to 8 and *ℓ* from 40 to 100 kbp. We note that in the error correction method we filter related Rmaps returned by the index based on the pairwise alignment. This additional filtering was not used in these experiments because it could confound the effect that the seeding methods have on precision and recall.

All experiments in this section were run on Intel Xeon E 5540 CPUs operating at 2.53 GHz, equipped with 32 GB of memory and running Linux 4.10. The running time and memory usage was recorded with the Linux/Unix time command. We report the elapsed (wall-clock) time. None of the indexing implementations take advantage of parallelism.

**Fig. 5.**
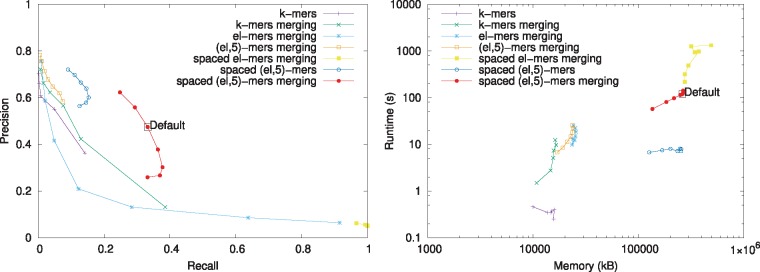
Comparison of the performance of the different indexing schemes. The precision and recall of the different indexing schemes when *k* or *ℓ* is varied is shown on the left and the runtime and memory usage of the different indexing schemes on the right. The performance of the spaced (*ℓ*, *k*)-mer index with the default parameters is shown with a black rectangle

The left hand side of Figure 5 shows the precision and recall for the different indexing schemes and the right hand side shows the trade-off between running time and memory. The *k*-mer index is the most efficient with respect to both memory and time but its recall is unsatisfactory. Merging similar *k*-mers improves the recall but significantly lowers the precision. Figure 5 (left) also illustrates that *ℓ*-mers are not selective. The spaced *ℓ*-mer index returns most Rmap pairs as related reflected by a low precision and a recall score of almost one. However, the (*ℓ*, *k*)-mers increase the precision substantively. For example, the spaced (ℓ,5)-mer index using merging has superior precision-recall trade-off but is less efficient with respect to memory and time than the *k*-mer index. Further, merging significantly improves the recall of the spaced (*ℓ*, *k*)-mer index with a small decrease in precision but it also increases the running time.

In light of these results, we ran experiments for various values of *k* for the spaced (*ℓ*, *k*)-mer index with merging of similar spaced (*ℓ*, *k*)-mers. Supplementary Figure S1 shows that *k *=* *5 gives the best trade-off and thus it was used in all other experiments.

Supplementary Figure S2 shows how the performance of the spaced (*ℓ*, *k*)-mer index is affected by the choice of the spacing pattern. The optimized spacing patterns perform better than the random ones although the difference is not large. Spacing patterns that have more weight in the beginning generally performed better. We also noticed that best spacing patterns can have 0s in the end. This is likely due to us adding fragments to the (*ℓ*, *k*)-mers until at least *k* fragments are used.

### 5.3 Performance of error correction

We compare the performance of our method Elmeri to our previous method, cOMet (Mukherjee *et al.*, 2018), on the full human and Ecoli1 datasets. Here, we performed all experiments on Intel E5-2698v3 processors with 192 GB of RAM running 64-bit Linux, and used the default parameters for cOMet. cOMet was parallelized to use five processes. Based on the experiments of the previous section, we set the spaced (*ℓ*, *k*)-mer index parameters for Elmeri as follows: *ℓ* = 80 kbp, *k *=* *5, S=11111111110001110110010010011101001110001010010100001010011000010111100000001100. The performance of the index with these default parameters is shown in Figure 5 with a black rectangle. Elmeri was run on a single node using eight threads for the error correction phase.

Elmeri obtained superior results on the human dataset when *k *=* *6, which is likely due to the increased size of the genome. Since the human genome is significant longer than *E.coli*, a larger value of *k* is likely needed to ensure most spaced (*ℓ*, *k*)-mers are unique in the genome. Further, since the human dataset has significantly lower coverage than each of the eight Ecoli1 datasets, we only consider the top 32 related Rmaps for each Rmap in the error correction algorithm instead of the default 64.

To evaluate the accuracy of correction, we aligned the uncorrected and the corrected Rmaps against an *in silico* digested reference genome using the alignment tool by Valouev *et al.* (2006a). As previously mentioned, the method of Valouev *et al.* (2006a) uses dynamic programming to find the optimal alignment for any pair of Rmaps by optimizing a scoring function that accounts for added and missing cut-sites, which is called the *S-score*. We then counted the number of Rmaps whose S-score had improved, and computed the mean increase in the S-score.

Table 2 shows the results on the Ecoli1 datasets. In all but one case, the percentage of Rmaps with improved S-score is higher for Elmeri than for cOMet. Furthermore, the mean increase in S-score is almost double for Elmeri as compared to cOMet, and Elmeri is significantly faster than cOMet. Elmeri uses more memory, however, all datasets were able to be ran with less than 21 GB of memory.

**Table 2.** The accuracy, runtime and memory usage of Elmeri and cOMet on simulated *E.coli* data when the number of additional cut sites introduced per 100 kbp and the rate of missing cut sites are varied

Added cut sites per 100 kbp	Deleted cut site rate	Percent of Rmaps with improved S-score		Mean increase in S-score		CPU time (hours)		Peak memory (GB)	
		Elmeri	cOMet	Elmeri	cOMet	Elmeri	cOMet	Elmeri	cOMet
0.5	15	93.22	**93.42**	**23.58**	12.46	**10.44**	24.50	13.67	**7.29**
1	5	**87.98**	87.36	**16.61**	6.30	**18.54**	35.85	16.11	**8.55**
	15	**94.79**	94.01	**24.97**	13.19	**11.90**	28.15	16.27	**7.56**
	25	**96.84**	96.09	**30.76**	17.20	**10.04**	42.20	15.68	**6.49**
2	5	**90.41**	89.23	**18.13**	7.93	**19.90**	55.01	20.20	**8.98**
	15	**93.68**	92.99	**24.20**	13.36	**16.78**	25.40	20.14	**7.29**
	25	**96.98**	93.02	**31.15**	14.70	**12.99**	66.01	19.46	**6.87**
5	15	**90.55**	81.35	**15.74**	6.71	**25.92**	143.15	29.52	**7.83**

*Note*: For each measured quantity we have highlighted the best result.

Table 3 shows the error correction results on the real human and *A.testudineus* Bionano data. A higher percentage of cOMet corrected Rmaps have an improved S-score but the mean S-score improvement of the Elmeri corrected Rmaps is more than 4 times that of cOMet on the human data and almost twice that of cOMet on the *A.testudineus* data. On the human data Elmeri is 16 times faster than cOMet but uses 5 times more memory, whereas on the *A.testudineus* data Elmeri is 34 times faster but uses 10 times more memory.

**Table 3.** The accuracy, runtime and memory usage of Elmeri and cOMet on human Bionano data and on *A.testudineus* genome

Dataset	Percent of Rmaps with improved S-score		Mean increase in S-score		CPU time (hours)		Peak memory (GB)	
	Elmeri	cOMet	Elmeri	cOMet	Elmeri	cOMet	Elmeri	cOMet
Human	69.21	**74.78**	**11.89**	2.69	**14.76**	236.80	101.39	**19.51**
AnaTes	59.15	**62.25**	**9.54**	5.00	**214.82**	7430.99	399.60	**37.61**

*Note*: For each measured quantity we have highlighted the best result.

To demonstrate the effect of error correction on assembling Rmap data, we corrected the Ecoli2 dataset with Elmeri and cOMet, then assembled the corrected Rmaps using the assembler of Valouev *et al.* (2006b). We aligned the assembled maps to the genome-wide optical map using the alignment method of Valouev *et al.* (2006a) and calculated the fraction of the genome covered by the assembled maps and the number of missing and added cut sites in the assembled map. Table 4 compares these assembled maps directly to the results presented by Mukherjee *et al.* (2018). The Rmaps corrected by Elmeri were assembled to a single map, whereas Rmaps corrected by cOMet assembled into two maps. The assembled maps from data corrected by Elmeri cover a larger fraction of the genome than assembled maps from cOMet corrected data and the number of missing and added cut sites in assembled maps produced from Elmeri corrected data is also less than third of those assembled from cOMet corrected Rmaps.

**Table 4.** Assembly results for uncorrected Rmaps, Rmaps corrected by cOMet and Elmeri and errorfree Rmaps

Rmap status	Number of assembled maps	Genome coverage	Missing/added cut sites
Uncorrected	5	81.2	47
Corrected by cOMet	2	82.2	34
Corrected by Elmeri	**1**	**86.2**	10
Error free	3	79.6	**1**

*Note*: The results for uncorrected Rmaps, Rmaps corrected by cOMet and errorfree Rmaps are directly from Mukherjee *et al.* (2018). We have highlighted the best results for each column.

## 6 Conclusion

Finding similar Rmaps is a fundamental step in many problems on optical mapping data such as finding pairwise alignments between Rmaps, aligning Rmaps against a reference and correcting errors in Rmaps. We have extended the notion of spaced seeds to optical mapping data by defining spaced (*ℓ*, *k*)-mers. We show that indexing spaced (*ℓ*, *k*)-mers more than doubles the recall for retrieving related Rmaps as compared to the previously introduced *k*-mer indexing.

We have also presented a simulated annealing based method for optimizing the spacing pattern. Further work in this direction includes studying the use of multiple spacing patterns as well as seed design in general. Many optimization techniques developed for spaced seeds in homology search, such as overlap complexity (Ilie and Ilie, 2007) and quadratic residual seeds (Egidi and Manzini, 2013), are likely applicable also in our setting. We apply spaced (*ℓ*, *k*)-mer based retrieval of related Rmaps to correcting Rmaps. We give results demonstrating that on a human dataset Elmeri is 16 times faster than cOMet, the only previous method for correcting Rmap data. We also show the alignment scores of Rmaps corrected by Elmeri improve more than four times on the scores for Rmaps corrected by cOMet. We note that Elmeri uses more memory than cOMet and suggest that the index structures used by Elmeri could be optimized to reduce the memory footprint. Lastly, we suggest that another direction for future work is to apply spaced (*ℓ*, *k*)-mers to other analysis problems that use optical mapping data, namely, Rmap alignment problems.

## Funding

This work was supported by Academy of Finland (grants 308030 and 314170 to L.S. and grants 294143 and 319454 to S.J.P.) and the National Science Foundation (NSF) IIS (Grant No. 1618814 to C.B., L.S. and S.J.P.).

*Conflict of Interest*: K.M. has done an internship at Bionano.

## Supplementary Material

btz663_Supplementary_File
