# Fast and accurate correction of optical mapping data via spaced seeds

**DOI:** 10.1093/bioinformatics/btaa101

**Published:** 2020-03-18

**Authors:** Leena Salmela, Kingshuk Mukherjee, Simon J Puglisi, Martin D Muggli, Christina Boucher

*Bioinformatics* (2019) doi:10.1093/bioinformatics/btz663

Incorrect supplementary material was published alongside the above manuscript. This has now been replaced with the correct material.

