# Supplementary material for: Fast and accurate correction of optical mapping data via spaced seeds
Source: Bioinformatics. 2019 Sep 3;36(3):682–9. doi: 10.1093/bioinformatics/btz663 (PMC7005598; doi:10.1093/bioinformatics/btz663)
Supplement: btz663_Supplementary_File [file btz663_supplementary_file.pdf]

Fast and Accurate Correction of Optical Mapping Data via Spaced Seeds

Supplementary material

Leena Salmela[1], Kingshuk Mukherjee[2], Simon J. Puglisi[1], Martin D. Muggli[3], and Christina Boucher[2]

[1]Department of Computer Science, Helsinki Institute for Information Technology HIIT, University of Helsinki, Helsinki, Finland
[2]Department of Computer and Information Science and Engineering, University of Florida, Gainesville, FL, USA
[3]Department of Computer Science, Colorado State University, Fort Collins, CO, USA.

1 Optimal k value for spaced (ℓ, k)-mer index

We ran experiments for various values of k for the spaced (ℓ, k)-mer index with merging of similar spaced (ℓ, k)-mers. Figure 1 shows the precision and recall for k equal to 4, 5, and 6. For $k = 5$ and $k = 6$, we considered two Rmaps related if they share at least two spaced (ℓ, k)-mers. Yet, when $k = 4$ the number of Rmap pairs sharing at least two spaced (ℓ, k)-mers was very high. Therefore, we obtained superior results by requiring that two Rmaps share at least five spaced (ℓ, k)-mers and thus, we used this threshold for the results in Figure 1. We see that $k = 5$ gives the best trade-off and thus, is used in all other experiments.

2 Optimal spacing patterns

Figure 2 shows how the performace of the spaced (ℓ, k)-mer index is affected by the choice of the spacing pattern. Results for the following spacing patterns are shown:

- S1: 11111111110001110110010010011101001110001010010100001010011000010111100000001100
- S2: 11110111111011001111000110101100111010110000001110100011010010100111110011000000
- S3: 1110110101001101011001001001010101100010
- S4: 11111111111111110001111111000011001100011100111100001110000000111101110000011000
- S5: 110000010101111010010000000011011111111111000110000101111100101110111010110001010
- S6: 111000100001111011010110000001000100001110101101101101111010110110001001001010110

Spacing pattern S1 is the default pattern which we used in all other experiments. Spacing pattern S2 is produced by the simulated annealing algorithm detailed in Section 4.6 optimizing the spacing pattern for $\ell = 80$ Kbp and $k = 5$. Spacing pattern S3 is also produced by the simulated annealing algorithm for $\ell = 80$ Kbp and $k = 5$ but the spacing pattern has length 40 where every bit represents a 2 Kbp region. Spacing pattern S4 is optimized for $\ell = 60$ Kbp and $k = 5$. Finally, spacing patterns S5 and S6 are generated randomly with equal probability for 0 and 1. The optimized spacing patterns perform better than the random ones although the difference is not large. The more fine grained 80-bit spacing patterns perform better than the 40-bit pattern S3. We noticed that spacing patterns that have more weight in the beginning generally performed better. We also noticed that best spacing patterns can have 0's in the end. This is likely due to us adding fragments to the (ℓ, k)-mers until at least k fragments are used.

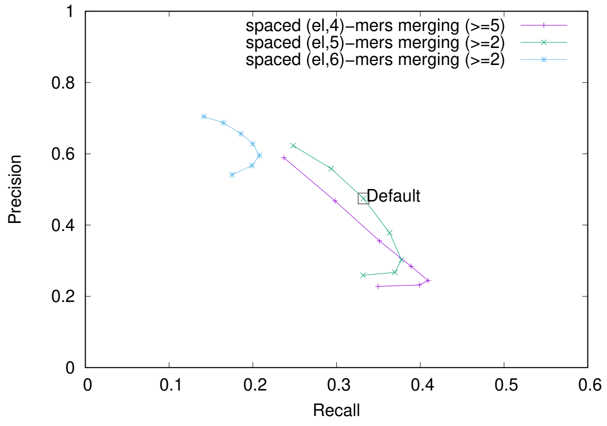

Figure 1: The precision and recall of the spaced (ℓ, k)-mer index for different values of k when ℓ is varied. For spaced $(\ell, 4)$-mers we show the performance when considering two Rmaps related if they share at least five spaced (ℓ, k)-mers. For spaced $(\ell, 5)$-mers and spaced $(\ell, 6)$-mers two Rmaps are considered related if they share at least two spaced (ℓ, k)-mers. The performance of the spaced (ℓ, k)-mer index with the default parameters is shown with a black rectangle.

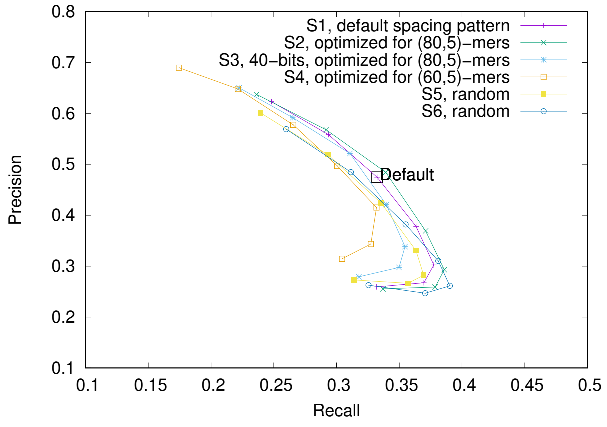

Figure 2: The precision and recall of the spaced $(\ell, 5)$-mer index for different spacing patterns when ℓ is varied. The performance of the spaced (ℓ, k)-mer index with the default parameters is shown with a black rectangle.

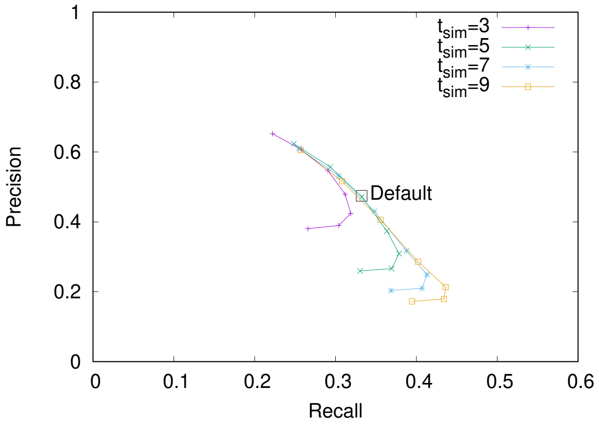

Figure 3: The precision and recall of the spaced $(\ell, 5)$-mer index for different values of the similarity threshold for merging $(\ell, 5)$-mers. The performance of the spaced (ℓ, k)-mer index with the default parameters is shown with a black rectangle.

3 Threshold for merging similar (ℓ, k)-mers

Firgure 3 shows how the performance of the (ℓ, k)-mer index varies when the threshold for merging similar (ℓ, k)-mers and ℓ is varied.
